# Mast Cell Accumulation in Glioblastoma with a Potential Role for Stem Cell Factor and Chemokine CXCL12

**DOI:** 10.1371/journal.pone.0025222

**Published:** 2011-09-19

**Authors:** Jelena Põlajeva, Anna M. Sjösten, Nina Lager, Marianne Kastemar, Ida Waern, Irina Alafuzoff, Anja Smits, Bengt Westermark, Gunnar Pejler, Lene Uhrbom, Elena Tchougounova

**Affiliations:** 1 Rudbeck Laboratory, Department of Immunology, Genetics and Pathology, Uppsala University, Uppsala, Sweden; 2 Smålandsvägen 11, Uppsala, Sweden; 3 Department of Anatomy, Physiology and Biochemistry, Swedish University of Agricultural Sciences, BMC, Uppsala, Sweden; 4 Department of Neuroscience, Uppsala University Hospital, Uppsala, Sweden; National Taiwan University Hosipital, Taiwan

## Abstract

Glioblastoma multiforme (GBM) is the most common and malignant form of glioma with high mortality and no cure. Many human cancers maintain a complex inflammatory program triggering rapid recruitment of inflammatory cells, including mast cells (MCs), to the tumor site. However, the potential contribution of MCs in glioma has not been addressed previously. Here we report for the first time that MCs infiltrate KRas+Akt-induced gliomas, using the RCAS/TV-a system, where KRas and Akt are transduced by RCAS into the brains of neonatal *Gtv-a-* or *Ntv-a* transgenic mice lacking *Ink4a* or *Arf*. The most abundant MC infiltration was observed in high-grade gliomas of *Arf−/−* mice. MC accumulation could be localized to the vicinity of glioma-associated vessels but also within the tumor mass. Importantly, proliferating MCs were detected, suggesting that the MC accumulation was caused by local expansion of the MC population. In line with these findings, strong expression of stem cell factor (SCF), i.e. the main MC growth factor, was detected, in particular around tumor blood vessels. Further, glioma cells expressed the MC chemotaxin CXCL12 and MCs expressed the corresponding receptor, i.e. CXCR4, suggesting that MCs could be attracted to the tumor through the CXCL12/CXCR4 axis. Supporting a role for MCs in glioma, strong MC infiltration was detected in human glioma, where GBMs contained significantly higher MC numbers than grade II tumors did. Moreover, human GBMs were positive for CXCL12 and the infiltrating MCs were positive for CXCR4. In conclusion, we provide the first evidence for a role for MCs in glioma.

## Introduction

Gliomas are the most frequent primary brain tumors of adults that are classified into grades I–IV based on malignancy. Glioblastoma multiforme (GBM) is the most common and malignant type of glioma that has poor prognosis, with a median survival time of just over one year [1] and no cure.

Hallmarks of glioma include disruption of the blood brain barrier (BBB) and aberrant invasiveness. Disruption of the BBB occurs in high-grade gliomas, is associated with abnormal neovasculature and extreme vessel leakiness, which promote expansion of GBM [2]. The process of invasion is an early and complex feature of glioma cells that is initiated already in low-grade gliomas and involves, in addition to glioma cell interactions with extra-cellular matrix (ECM), multiple additional factors accompanying glioma cell movement [3].

The rate of development and growth of tumors is regulated by the balance between pro- and anti-tumorigenic signals, produced either by the tumor cells themselves, or by the surrounding microenvironment. Local chronic inflammation, at the site of tumor growth, is a potent cancer promoter and results in induction of angiogenesis, tissue remodeling and immune modulation. Many human cancers, including gliomas, instruct and maintain a complex inflammatory program that, among other effects, triggers rapid recruitment of inflammatory cells to the tumor site. For example, immune infiltration of gliomas was recognized as one of the processes following the development of advanced gliomas, and it has been demonstrated that gliomas are infiltrated by microglia, CD4 and CD8 T lymphocytes, and natural killer (NK) cells [4], [5]. Generally, there appears to exist a positive correlation between the extent of immune infiltration and poor clinical outcome. However, the exact contribution of the immune system in tumorigenesis is still not clear.

Mast cells (MCs) are crucial players in various inflammatory conditions, including cancer [6]. The specific role of MCs in tumorigenesis may vary largely, depending on the type of cancer. MCs have been identified as an early highly infiltrative cell type in skin dysplasias [7], breast carcinoma [8], colorectal carcinoma [9], malignant melanomas [10], and pancreatic islet tumors [11]. A protumorigenic role for MCs has been indicated in thyroid cancer [12] and MCs have been associated with poor prognosis in prostate cancer [13]. Conversely, an association between presence of MCs and improved prognosis has also been documented [14]. Hence, MC-related inflammatory processes might either facilitate or hinder cancer, depending on the type of tumor setting. Notably, previous studies investigating the role of MCs in cancer have been focused on tumors outside of the central nervous system (CNS). Here we are for the first time analyzing the potential involvement of MCs in brain tumors. MCs are known to populate the CNS of several species, including humans and have been found there from the time of birth. In the brains of mammals they are concentrated in the medial habenula (MHb), which is part of the epithalamus [15].

During the past decade, the RCAS/TV-a mouse model has become an important system for studying glioma [16]. RCAS/TV-a allows post-natal gene transfer mediated by oncogene-carrying RCAS retrovirus into brains of transgene animals engineered to express the *tv-a* receptor under the control of cell-type-specific promoters (e.g. *Ntv-a*; *Gtv-a*). In *Ntv-a* mice, the nestin promoter directs infection to neural/glial progenitor cells, while in *Gtv-a* mice, the glial fibrillary acidic protein (GFAP) promoter directs infection mainly to astrocytes. The *tv-a* transgenic mice have been cross-bred with mice carrying targeted deletions of tumor suppressor genes (*Ink4a−/−*; *Arf−/−*) frequently deleted in human glioma [17]. Tumors can be induced by RCAS-mediated transfer of oncogenes, e.g. *PDGF*, *Kras*, *Akt* that closely resembles the histopathology and genetics of human glioma. Using such models, key mechanistic insights into gliomagenesis have been obtained [17], [18], [19], [20]. RCAS/TV-a experimental models have also been used for pre-clinical testing of immunotherapies [21]. In the present investigation we have used gliomas generated from *Ntv-a* and *Gtv-a* transgenic mouse lines by transduction of Kras and Akt [17].

We have investigated whether MCs have a role in glioma and show for the first time that both mouse and human gliomas accommodate MCs. Moreover, we present evidence suggesting that tumor-associated vessels produce SCF that drives MC proliferation and that MC chemotaxis in gliomas involves interaction between CXCL12 and CXCR4.

## Results

### Infiltration of connective tissue type mast cells in mouse glioma

**Figure 1:**
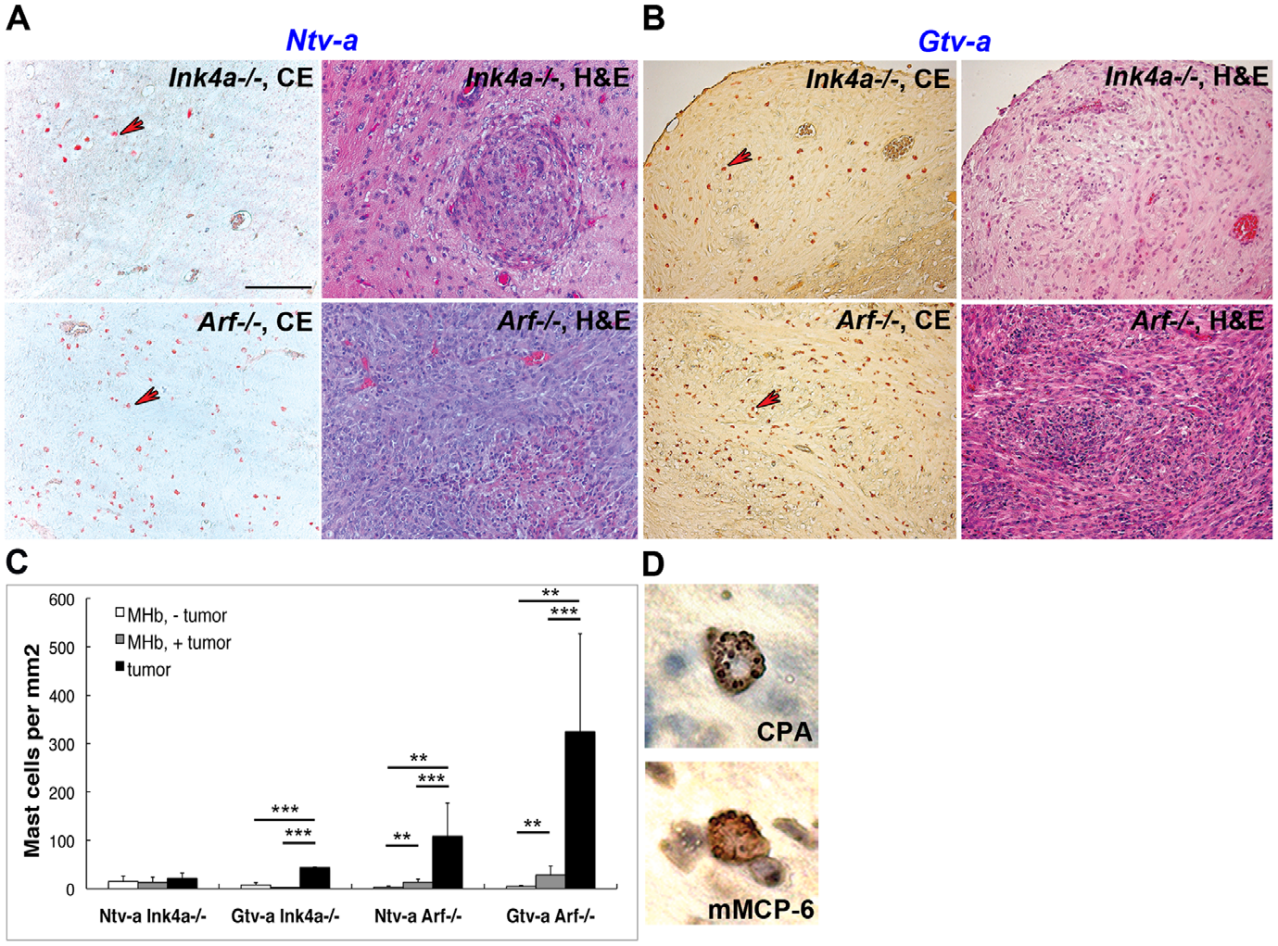
Accumulation of MCs in RCAS-*KRas*+RCAS-*Akt* induced tumors from *Ntv-a Ink4a−/−*, *Ntv-a Arf−/−*, *Gtv-a Ink4a−/−* and *Gtv-a Arf−/−* mice. (**A**) Chloroacetate esterase (CE) and H&E-stained *Ntv-a Ink4a−/−* and *Ntv-a Arf−/−* tumors. Arrows in left panels indicate MCs. Scale bar = 100 µM. (**B**) Chloroacetate esterase (CE)- and H&E-stained *Gtv-a Ink4a−/−* and *Gtv-a Arf−/−* tumors. Arrows indicate MCs. (**C**) Quantification of MCs in both non-tumor MHb (MHb, − tumor), cancerous MHb (MHb, + tumor) and in the tumor area (tumor) of mouse glioma samples. *Ntv-a Ink4a−/−* (MHb − tumor, n = 6; MHb + tumor, n = 12; tumor, n = 12), *Ntv-a Arf−/−* (MHb − tumor, n = 6; MHb + tumor, n = 12; tumor, n = 12), *Gtv-a Ink4a−/−* (MHb − tumor, n = 6; MHb + tumor, n = 2; tumor, n = 2), *Gtv-a Arf−/−* (MHb − tumor, n = 6; MHb + tumor, n = 12; tumor, n = 12) revealing statistically significant difference between all compared groups for *Arf* deficient mice (** p<0.01, *** p<0.001). Error bars show SD. (**D**) MC carboxypeptidase A (MC-CPA)- and mMCP-6 positive MCs from the *Ntv-a Arf−/−* tumor.

To address the potential role for MCs in gliomagenesis, we used archival RCAS/TV-a derived gliomas of various grades and types. In previous studies it has been shown that the absence of either of the key tumor suppressors *Ink4a* or *Arf* in *Ntv-a* and *Gtv-a* transgenic mice yields tumors upon infection with the combination of RCAS-*KRas* + RCAS-*Akt*. *Arf*-loss caused increased malignancy of gliomas compared to *Ink4a*-loss, indicating a prominent role for *Arf* in tumor progression [17]. Tumors were analyzed for the presence of MCs by performing chloroacetate esterase enzymatic staining, which detects chymotrypsin-like activity within MC granules. As shown in Figure 1, MCs were present in gliomas of both *Ntv-a* and *Gtv-a* transgenes, and both in *Arf−/−* and *Ink4−/−* animals. However, the most profound MC infiltration was seen in high-grade gliomas, i.e. in *Arf−/−* mice, whereas less prominent MC infiltration was seen in low-grade gliomas, i.e. in *Ink4a−/−* mice (Figure 1A–B). Quantification of these data revealed that in addition to striking increase of MC numbers in glioma area, there was also a significant increase in MC numbers within glioma-associated MHb in *Arf* knock-out mice (Figure 1C). Hence, MCs infiltrate gliomas, with the most evident MC infiltration found in high-grade gliomas in *Arf−/−* mice. Therefore, in the subsequent experiments we have focused on tumors formed in *Arf−/−* mice.

There are two main subtypes of MCs in mouse, connective tissue type MCs (CTMCs) and mucosal type MCs (MMCs), and these are distinguished by characteristic expression profiles of various MC-specific proteases [22]. CTMCs express predominantly chymases of the mouse mast cell protease 4 (mMCP-4) and mMCP-5 types, tryptases mMCP-6 and mMCP-7, and MC carboxypeptidase A (MC-CPA), whereas MMCs express the chymases mMCP-1 and mMCP-2 but no tryptases or MC-CPA. As shown in Figure 1D, MCs present in tumors of *Ntv-a*- and *Gtv-a Arf−/−* mice stained positively for both of the CTMC markers, mMCP-6 and MC-CPA, but were negative for the MMC marker mMCP-1 (not shown). Hence, MCs in the experimental gliomas were of the CTMC subtype.

### Frequent perivascular localization of mast cells in mouse gliomas

**Figure 2:**
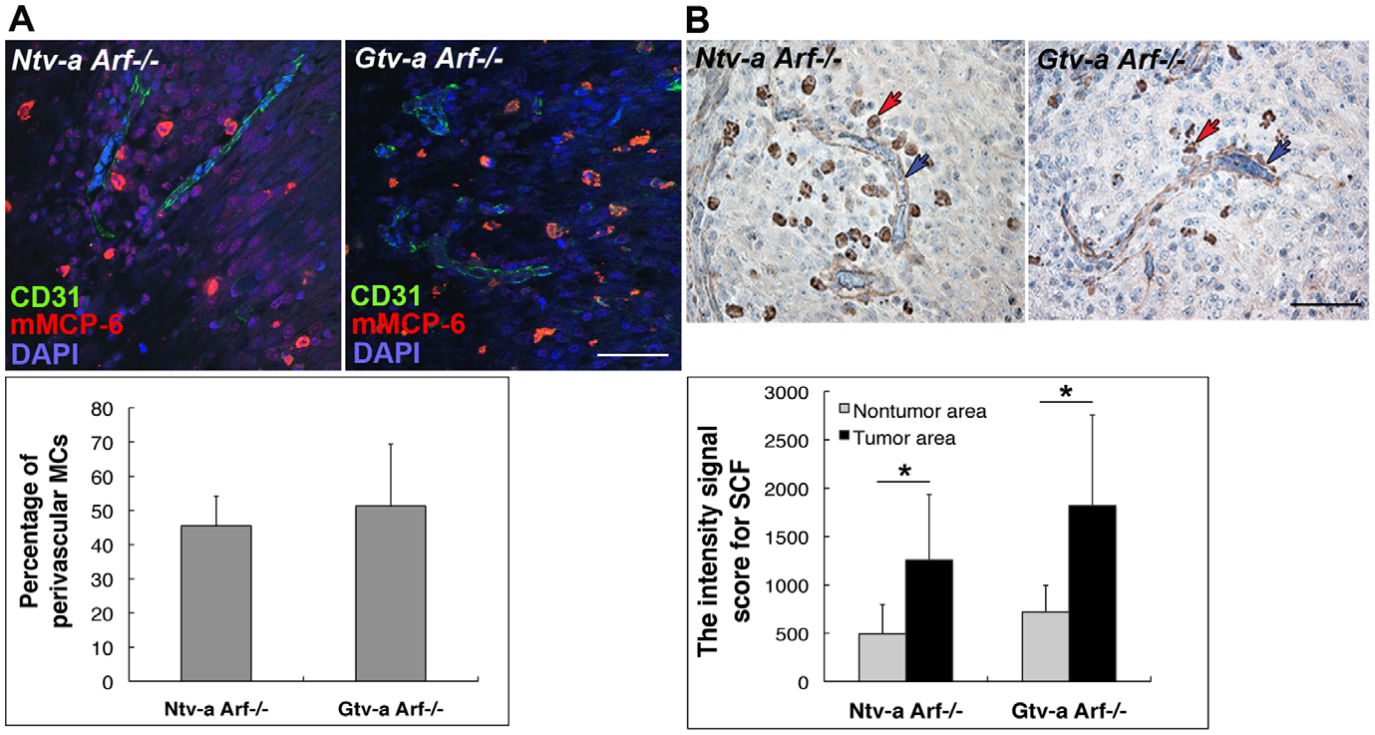
MC distribution in the mouse RCAS-*KRas*+RCAS-*Akt* induced brain tumors. (**A**) Immunofluorescence staining for endothelial cell marker CD31 and MC tryptase mMCP-6 in *Ntv-a Arf−/−* and *Gtv-a Arf−/−* mouse brain tumors revealed perivascular localization of MCs. Lower panel: quantification of perivascular MCs in mouse brain tumors revealed about 50% in the corresponding objective fields with no difference between *Ntv-a Arf−/−* (n = 5) and *Gtv-a Arf−/−* (n = 5). Error bars show SD. Scale bar = 50 µM. (**B**) Immunohistochemical analysis for SCF expression revealing marked expression of SCF in the glioma vascular structures in both *Ntv-a Arf−/−* and *Gtv-a Arf−/−* mice (indicated by blue arrows). Expression of SCF was also observed in MC granules (indicated by red arrows). Lower panel: quantification of total absolute intensity signal for SCF revealed statistically significant difference between tumor and nontumor areas of the objective fields in both *Ntv-a Arf−/−* and *Gtv-a Arf−/−* mouse brain tumors (* p<0.05). Error bars show SD. Scale bar = 50 µM.

As shown by co-staining for the endothelial cell marker CD31 and the MC marker mMCP-6, MCs within experimental gliomas frequently showed a perivascular localization (with an average of 50% for both *Ntv-a-* and *Gtv-a Arf−/−* mice) (Figure 2A), but were also present within the tumor mass in both *Ntv-a-* and *Gtv-a* lines.

SCF is the most important growth factor for MCs in all species [23]. SCF is an essential chemoattractant for MCs that controls differentiation of MCs and induces MC proliferation and degranulation. We reasoned that the massive increase in MC numbers in the high-grade gliomas, as opposed to normal tissue, might be a result of glioma-driven SCF expression. We analyzed the expression of SCF in the mouse gliomas and found significantly increased expression of SCF in tumor vessels of both *Ntv-a*- and *Gtv-a Arf−/−* mice (Figure 2B, blue arrow) but also MC granules were positive (Figure 2B; indicated by red arrows). In contrast, SCF expression in vessels of non-tumor tissue was poor. Thus, KRas+Akt-induced experimental gliomagenesis is closely associated with increased, predominantly vascular expression of SCF, providing a potential explanation for the increased MC numbers in the tumor tissues.

### Proliferation of mast cells in mouse glioma

**Figure 3:**
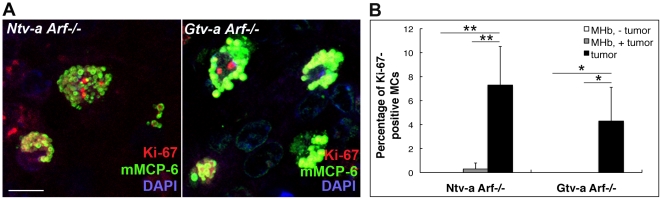
Proliferation of MCs in mouse RCAS-*KRas*+RCAS-*Akt* induced gliomas. (**A**) Immunofluorescence co-staining for proliferation marker Ki-67 and MC tryptase mMCP-6 in mouse brain tumors, revealing proliferation of MCs in both *Ntv-a Arf−/−* and *Gtv-a Arf−/−* mouse gliomas. Maximum intensity projection of z-stack confocal images was applied. (**B**) Quantification of proliferative MCs in both non-tumor MHb (MHb, − tumor), cancerous MHb (MHb, + tumor) and in the tumor area (tumor) of mouse glioma samples. The number of Ki-67-positive MCs in the tumor was significantly higher as compared to both MHb controls, being 7% and 4% in *Ntv-a Arf−/−* and *Gtv-a Arf−/−* mouse gliomas, respectively. For both *Ntv-a Arf−/−* and *Gtv-a Arf−/−* mouse gliomas: MHb − tumor, n = 6; MHb + tumor, n = 6; tumor, n = 6; * p<0.05, ** p<0.01. Error bars show SD.

The accumulation of MCs in gliomas may be the result of increased recruitment of blood-borne MC progenitors. An alternative explanation could be that the increase in MC numbers is a result of proliferation of local MC populations. To evaluate this latter possibility we analyzed for the presence of proliferating MCs, by doing double staining for Ki-67 (a marker for proliferating cells) and mMCP-6 (a marker of differentiated MCs). Indeed, we were able to identify proliferating MCs in both *Ntv-a-* and *Gtv-a Arf−/−* mouse gliomas as compared with non-tumor tissue (Figure 3A), with an average of 7% and 4% of proliferative MCs, respectively (Figure 3B). The double positivity for Ki-67 and mMCP-6 suggested that the gliomas could induce proliferation of functional MCs. Since no validated markers for MC progenitors have been established, we were not able to evaluate the possibility that the gliomas induced proliferation of local MC progenitors.

### Evidence for an active CXCL12/CXCR4 axis in mouse glioma

**Figure 4:**
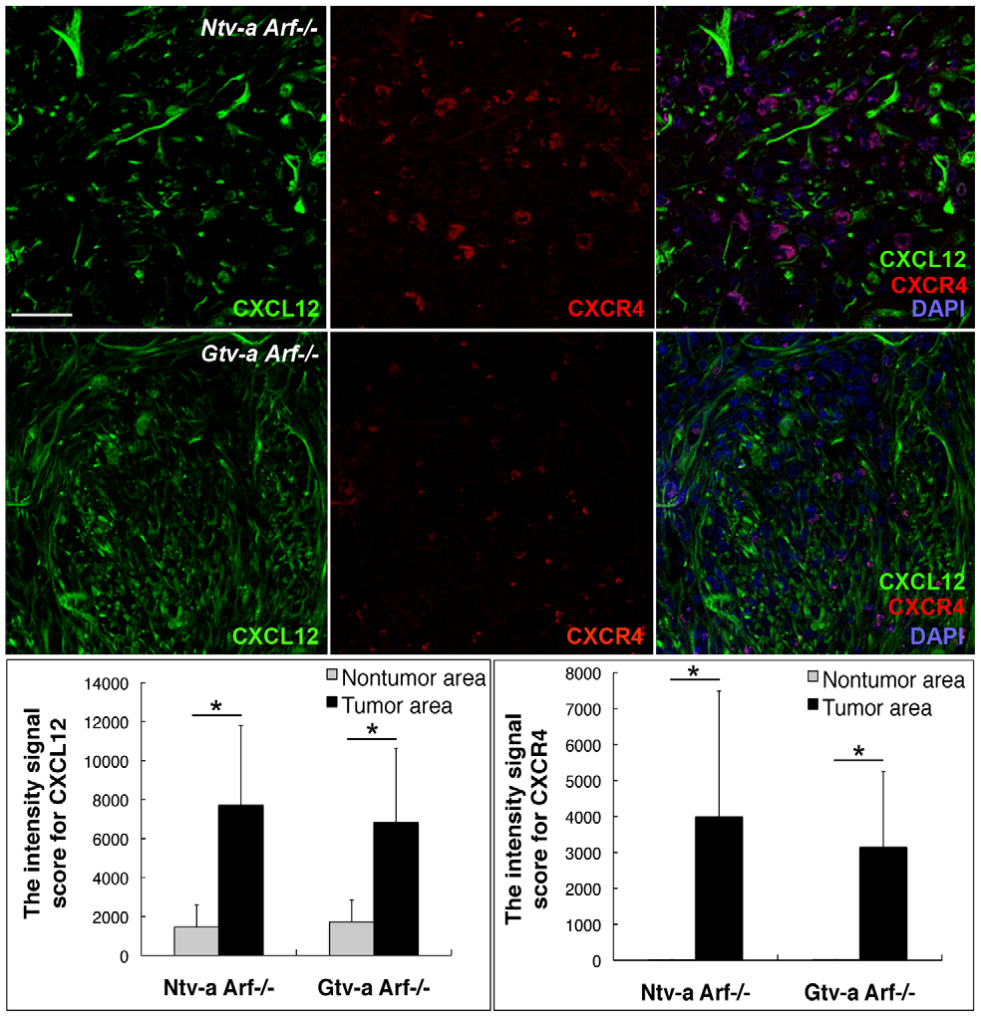
CXCL12 and CXCR4 expression in mouse RCAS-*KRas*+RCAS-*Akt* induced gliomas. Immunofluorescence staining for CXCL12 and CXCR4 was performed in both *Ntv-a Arf−/−* and *Gtv-a Arf−/−* mouse gliomas. The quantification of intensity signal for CXCL12 (lower left panel) and CXCR4 (lower right panel) revealed statistically significant difference between tumor and nontumor areas of the objective fields in both *Ntv-a Arf−/−* and *Gtv-a Arf−/−* mouse brain tumors (* p<0.05). Error bars show SD. Scale bar = 50 µM.

**Figure 5:**
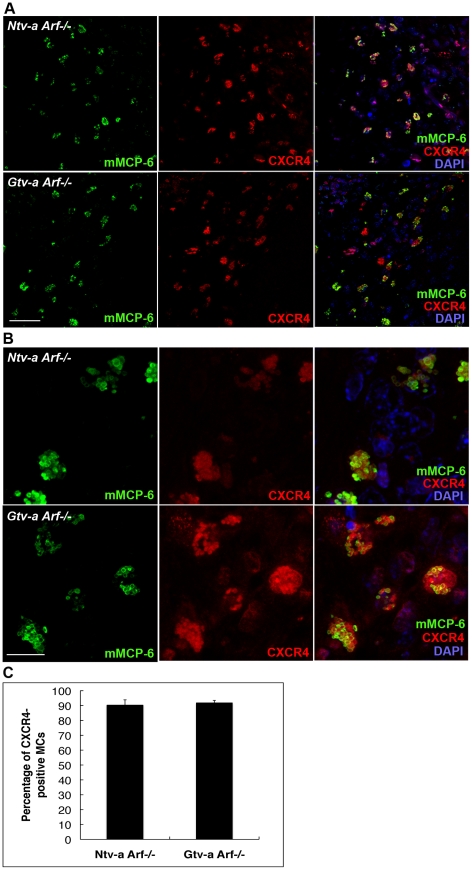
Co-expression of CXCR4 and mMCP-6 by MCs in mouse RCAS-*KRas*+RCAS-*Akt* induced gliomas. (**A**) Immunofluorescence staining for CXCR4 and mMCP-6 was performed in both *Ntv-a Arf−/−* and *Gtv-a Arf−/−* mouse gliomas, demonstrating co-expression of CXCR4 and mMCP-6. Scale bar = 50 µM. (**B**) Immunofluorescence staining, demonstrated co-localization of CXCR4 and mMCP-6 at the single-cell level in both *Ntv-a Arf−/−* and *Gtv-a Arf−/−* mouse gliomas. Maximum intensity projection of z-stack confocal images was applied. Scale bar = 20 µM. (**C**) The quantification of MCs in mouse brain tumors revealed about 90% to be CXCR4-positive in the corresponding objective fields with no difference between *Ntv-a Arf−/−* (n = 5) and *Gtv-a Arf−/−* (n = 5). Error bars show SD.

The chemokine CXCL12 (also known as stromal cell derived factor-1 (SDF-1)) is widely expressed in many tissues throughout development [24] and serves as a powerful chemoattractant for hematopoietic cells, facilitating their migration through endothelial cell barriers [25]. Moreover, CXCL12 is expressed in a large number of tumors and injured tissues, and the corresponding activation of its receptor, CXCR4, promotes angiogenesis [26] and metastasis of tumor cells [27]. Since CXCL12 is also known to act as a chemoattractant for MCs [28], we reasoned that MC migration into the tumor tissues might be the result of an interaction between CXCL12 and CXCR4. It has been previously shown, that CXCL12 was expressed by glioma cells [29], [30] and its expression increased with increasing tumor grade. We analyzed gliomas for expression of CXCL12 and CXCR4 and found abundant expression of CXCL12 in both *Ntv-a-* and *Gtv-a Arf−/−* gliomas (Figure 4; left panel) and there was also a clear expression of CXCR4 in the same samples (Figure 4; middle panel). CXCL12 expression was confined to the tumor stroma and very weak expression in the surrounding normal tissue could be found. In agreement with a role of the CXCL12/CXCR4 axis in promoting MC migration into the tumors, mMCP-6 positive MCs in both *Ntv-a Arf*−/− and *Gtv-a Arf*−/− mouse gliomas were frequently (with an average of 90%) CXCR4 positive (Figure 5A–C). Interestingly, the staining for mMCP-6 and CXCR4 revealed a spatial colocalization in MCs, suggesting that part of the CXCR4 pool could be present at the same site as mMCP-6 (secretory granules), but also that a fraction of the CXCR4 protein is present at sites distinct from the granules (Figure 5B; right panels).

**Figure 6:**
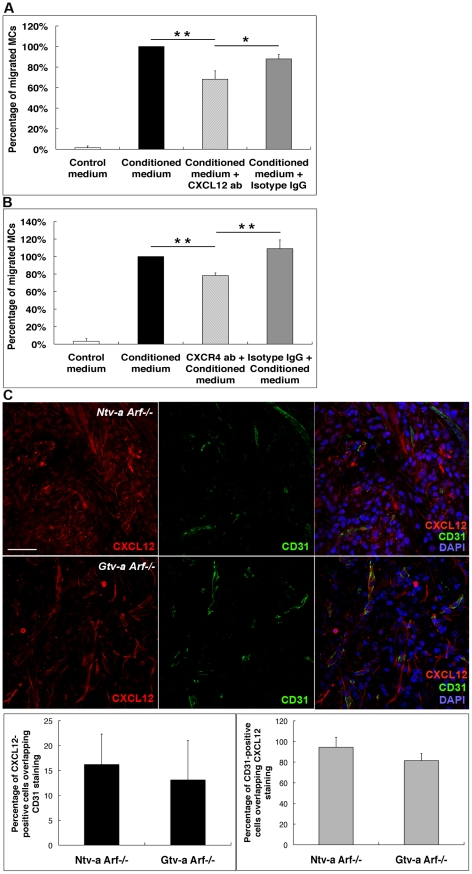
Demonstration of MC migration toward glioma-conditioned medium. Co-expression of CXCL12 and CD31 in mouse RCAS-*KRas*+RCAS-*Akt* induced gliomas. (**A**) Trans-well assay using CXCL12-neutralizing antibodies revealed statistically significant decreased migration of BMMCs towards glioma-conditioned medium. (**B**) Trans-well assay using antibodies to block CXCR4 receptor expressed on the BMMC surface demonstrated statistically significant decrease in BMMC migration towards glioma-conditioned medium. Appropriate isotype controls were used (* p<0.05, ** p<0.01). (**C**) Immunofluorescence staining demonstrated co-localization of CXCL12 and CD31 in both *Ntv-a Arf−/−* and *Gtv-a Arf−/−* mouse gliomas. Image analysis revealed an average of 16% and 14% of total CXCL12-positive cells in *Ntv-a Arf−/−* and *Gtv-a Arf−/−* mouse gliomas respectively were co-localized with CD31-positive endothelial cells. However, almost 94% and 82% of total CD31-positive cells in *Ntv-a Arf−/−* and *Gtv-a Arf−/−* mouse gliomas correspondingly were co-localized with CXCL12-positive staining. No statistical difference between *Ntv-a-* and *Gtv-a* lines was found. Scale bar = 50 µM. Error bars show SD.

To elucidate the mechanistic interplay of the receptor-bearing and ligand-producing cells, we performed an *in vitro* migration assay in which bone marrow derived mast cells (BMMCs) were placed into hanging inserts and were allowed to actively migrate through a porous membrane towards conditioned medium acquired after a 72 hour long glioma cell culturing period. A glioma cell line was established from a *KRas*+*Akt*-induced mouse tumor. Migration of untreated MCs towards glioma cell-conditioned medium was set to 100% and unconditioned medium was used as a negative control. Specific blockade of CXCL12 decreased MC migration by circa 32% and 20% as compared to migration towards glioma cell-conditioned medium or glioma cell-conditioned medium supplemented with non-specific IgG, respectively (Figure 6A). Similar results were obtained when CXCR4 was specifically blocked on the MC surface and cells were allowed to migrate towards glioma-conditioned medium (Figure 6B). Furthermore, in accordance with a number of previous studies [29], [31], endothelial cells can be an additional source of CXCL12. We therefore analyzed the gliomas for expression of CXCL12 and the endothelial cell marker CD31 (Figure 6C). The quantification revealed that up to 95% of endothelial cells are indeed CXCL12 positive in both *Ntv-a Arf*−/− and *Gtv-a Arf*−/− mouse gliomas (Figure 6C). However, only 15% of all CXCL12-positive cells are endothelial cells. This suggests that glioma cells, in accordance with the results from the MC migration assay, are the primary source of CXCL12 production and hence attract MCs via a CXCL12/CXCR4 axis.

### CXCL12 is a potential chemotaxin for CXCR4-positive MCs in human GBM

**Figure 7:**
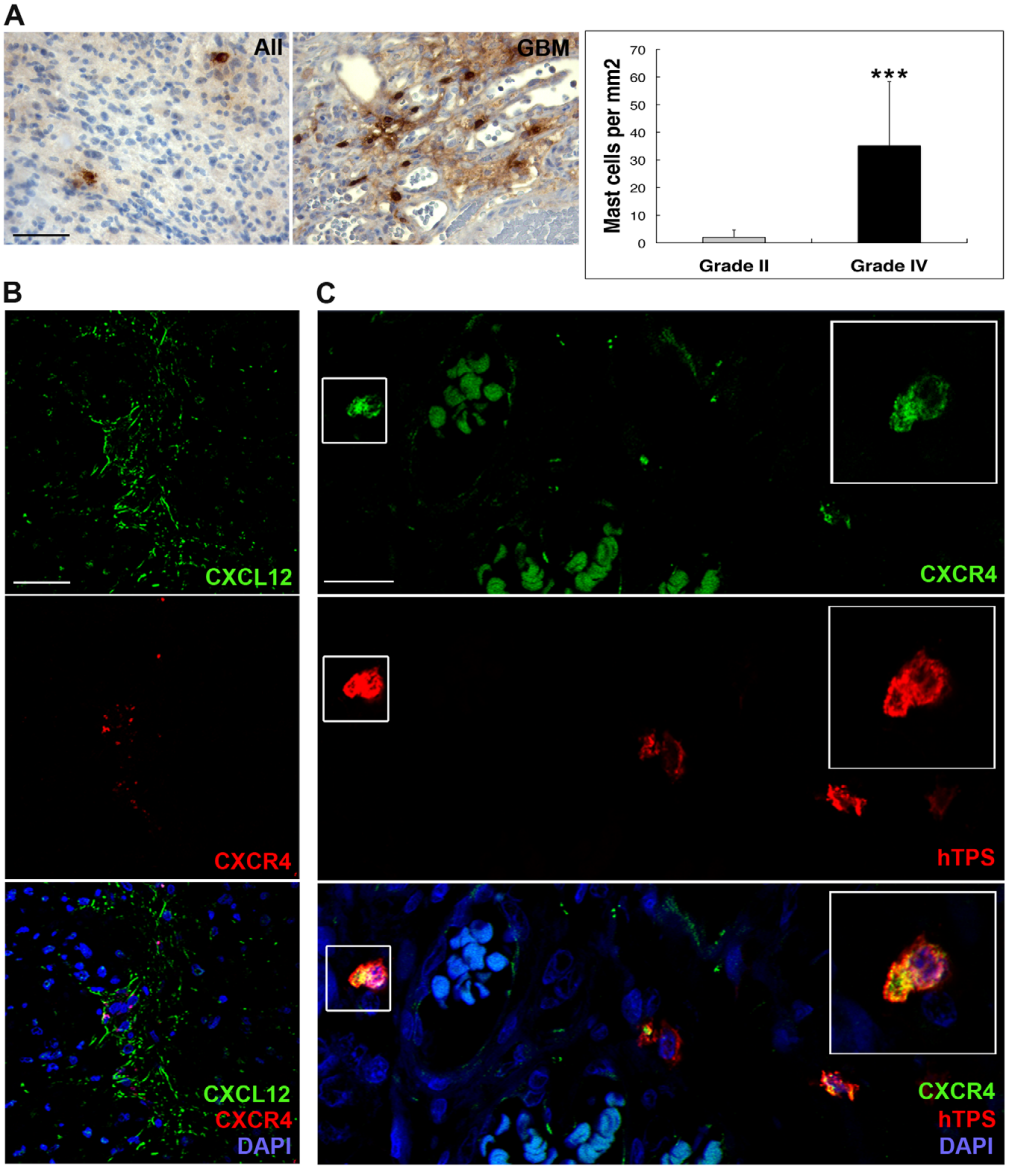
MC infiltration of human gliomas. (**A**) Immunohistochemical analysis of human MC tryptase (hTRS) in human low-grade gliomas (grade II, n = 8) and glioblastomas multiforme (GBM) (grade IV, n = 10). Right panel: quantification of MCs. Error bars show SD, *** p<0.001. Scale bar = 50 µM. (**B**) Immunofluorescence staining for CXCL12 and CXCR4 in human GBMs. Scale bar = 50 µM. (**C**) Immunofluorescence staining for CXCR4 and hTPS in human GBMs displayed co-expression of CXCR4 and hTPS. Scale bar = 25 µM. The inset represents a MC with co-localization of CXCR4 and hTPS at the single-cell level where maximum intensity projection of z-stack confocal images was applied.

In order to evaluate the potential role of MCs in human gliomas, we stained human low-grade gliomas (II) and GBMs (grade IV) for MC tryptase (hTPS). A comparison of the grade II and grade IV tumors demonstrated a remarkable accumulation of MCs in the more malignant tumors (GBMs) (n = 10) as compared to grade II tumors (n = 8) (Figure 7A).

All GBM patients used in the study had received glucosteroids prior to and after surgery (Table 1). This anti-inflammatory drug is known to have an indirect effect on reduction of MC numbers *in vivo* [32]. Quantification of positive cells revealed a statistically significant difference (p<0.001) in MC numbers between low-grade and high-grade gliomas (Figure 7A), and suggests that MC accumulation accompanies development of GBM in humans (Figure 7A). Similar to mouse gliomas, MCs in the human gliomas showed a predominantly perivascular localization and stained positive for both CXCL12 and CXCR4 (Figure 7B). Further, mimicking the situation in the mouse gliomas, tryptase-positive MCs present in human GBMs were frequently positive for CXCR4 (Figure 7C).

**Table 1:** Summary of patient characteristics and treatment received.

Patient	Histology Clinical type	Tumor location	Gender	Age at operation, year	Glucosteroids prior and post- operation
1	OII	Central lobe	F	33	No
2	OII	Frontal lobe	M	51	No
3	OII	Frontal lobe	M	42	No
4	AII	Frontal lobe	F	69	No
5	AII	Frontal lobe	F	48	No
6	AII	Tempoparietal lobe	F	36	No
7	AII	Temporal lobe	F	36	No
8	OII	Temporal lobe	F	34	No
1	Primary GBM	Frontal lobe	F	71	Yes
2	Primary GBM	Frontal lobe	F	59	Yes
3	Primary GBM	Temporal lobe	F	46	Yes
4	Primary GBM	Frontal lobe	M	46	Yes
5	Primary GBM	Frontal lobe	M	68	Yes
6	Primary GBM	Right tempoparietal lobe	F	64	Yes
7	Primary GBM	Right tempoparietal lobe	M	66	Yes
8	Primary GBM	Frontal lobe	F	69	Yes
9	Primary GBM	Frontal lobe	F	56	Yes
10	Primary GBM	Temporal lobe	F	41	Yes

OII = oligodendroglioma grade II, AII = astrocytoma grade II, Primary GBM = primary glioblastoma, F = female, M = male.

## Discussion

It still remains uncertain whether inflammation is the cause or the result of cancer. Despite numerous studies addressing this issue, the genuine connections between inflammatory- and tumor cells are still unresolved. Glioma is one of the types of cancer with most discouraging prognosis, and research on glioma has therefore expanded dramatically. However, the contribution of inflammation in gliomagenesis is not fully understood. Previous studies have implicated various immune cells, such as T cells, microglia and NK cells, during glioma development. Generally, these types of immune cells have been suggested to have pro-tumorigenic effects, with their presence being correlated with increased malignancy grade [4], [5].

During the past years, a number of studies showing significant correlations between MC infiltration and cancer development have been published [8], [11], [12], [13]. In many cases, the presence of MCs has been correlated with poor prognosis, but associations of MCs with improved prognoses have also been documented [14]. Thus the prognostic value of MC infiltration into tumors is controversial, and the exact role of MCs in tumor development remains elusive. In fact, it cannot be excluded that MCs perform partly opposing roles in tumor formation/development during distinct tumor stages, this being in line with the proposed protective effect of the immune system in early phases of tumorigenesis while the immune system may enhance tumorigenesis at later stages of tumor development [33].

Here we have expanded our understanding of the role of inflammation in gliomas by showing, for the first time, that MCs infiltrate mouse and human glioma, and that the extent of MC infiltration, both in mouse and human gliomas, shows a strong positive correlation with the malignancy grade of the tumor. Despite the pre- and post-treatment of GBM patients with glucosteroids, the difference in MC numbers was significant as compared to low-grade samples (not receiving the anti-inflammatory drug), and suggests that it could have been even more pronounced. The mouse studies were performed using the RCAS/TV-a mouse model system that produces life-like gliomas of different types and grades. We compared the extent of MC infiltration in tumors, induced with a combination of two oncogenes (*KRas+Akt)* in two different transgenic lines (*Ntv-a* and *Gtv-a*), each carrying a deficiency in either of the tumor suppressors *Ink4a* or *Arf*. We found that the number of MCs in high-grade tumors generated in *Arf-*deficient mice was significantly higher than in low-grade gliomas formed in *Ink4a*-deficient mice, thus suggesting that accumulation of MCs accompanies development of high-grade gliomas. In contrast, the cell-of-origin, i.e. neural/glial cell progenitors (*Ntv-a*) or astrocytes (*Gtv-a*) did not affect the extent of MC accumulation to any significant extent, indicating that MCs are accumulated in gliomas regardless of the cellular origin of the tumor. Notably, the induction of tumors by *KRas+Akt* in *Arf−/−* mice has been shown to generate predominantly GBM-like gliomas. By using this model, we have been able to get a more complete understanding of the processes leading to MC infiltration and distribution during glioma progression, than by mere examination of human glioma material.

The pronounced accumulation of MCs in the mouse gliomas suggested that glioma cells produce factors that can stimulate proliferation of MCs, as well as attracting MCs to migrate into the tumor. We show that SCF was highly expressed in tumor blood vessels but not in vessels outside the tumor tissue, and propose that the accumulation and perivascular localization of MCs in gliomas is, at least partly, explained by a glioma-driven induction of SCF expression. This hypothesis is also supported by a previous study, in which SCF expression was demonstrated in blood vessels of human glioma [34]. Another striking finding was that MCs themselves were strongly positive for SCF, arguing that expansion of the brain tumor-associated MCs may result, at least partly, from an autocrine loop induced by release of MC-contained SCF, which subsequently could bind to its receptor (c-kit) on the MC surface. Binding of SCF to c-kit could then induce MC proliferation, and, in agreement with this, we observed proliferation of glioma-associated MCs. Together, these data demonstrate for the first time that brain tumor-associated MCs contain endogenous sources of SCF, a finding that is in agreement with a previous study showing that human skin MCs may contain a preformed pool of SCF [35].

SCF may, in addition to inducing MC proliferation, also serve as a MC chemoattractant. However, SCF expression was low within the tumor mass, and the localization of MCs within the tumor mass was therefore most likely the result of chemotaxis induced by factors other than SCF. Taking into account that the CXCL12/CXCR4 interaction has a pronounced role in development of tumor vasculature [36], and that CXCL12 is a known MC chemotaxin [28], we demonstrated that both mouse and human gliomas were highly positive for CXCL12. Further, human and mouse glioma MCs were strongly positive for its cognate receptor CXCR4, this being in agreement with previous reports showing the expression of CXCR4 in human glioma [36], [37]. In the *in vitro* migration assay CXCL12 neutralization in glioma-conditioned medium led to a significant decrease of MC migration. Similarly, blocking of its receptor, CXCR4, resulted in reduced migration of MCs further, strengthening the notion that a CXCL12/CXCR4 axis plays an active role in MC recruitment to the tumor site. Hence, we propose that the MC accumulation within the gliomas is, at least partly, explained by a glioma-driven expression of CXCL12, combined with strong expression of CXCR4 within the brain tumor MC population.

CXCL12/CXCR4 interactions have been implied in vascularization processes, and it has been demonstrated that CXCL12 signaling is inducible in pathological conditions of the CNS [38] pointing to a potential role of CXCL12 in pathological formation of vessels within the brain. Accordingly, it has been suggested that the CXCL12/CXCR4 axis modulates the formation of new vessels under certain pathological conditions of the brain [39]. Despite being expressed by glioma cells at a higher level, CXCL12 is additionally expressed by endothelial cells further increasing its chemotactic potential.

The findings presented here show that MCs are present in glioma with higher numbers in more malignant tumors proposing a potential prognostic value for detecting MCs. The result suggests that MCs contribute to the progression of glioma but the exact nature of this contribution remains to be elucidated. One favored possibility would be that MCs promote the tumor angiogenesis in gliomas, a notion that is well in line with a proposed function of MCs in various other types of tumor settings [33]. Potentially, MCs may promote angiogenesis by multiple mechanisms, including the secretion of angiogenic factors [40] or by the secretion of MC proteases that may either directly degrade ECM components or promote ECM degradation by activating other ECM-degrading proteases such as matrix metalloprotease 9 [41]. There is as yet no cure for high-grade gliomas. Since MCs are normal cells within the tumor that should be less sensitive to developing therapy resistance, potential drugs that modulate MC driven processes in glioma may be a valuable complement to other treatments.

## Materials and Methods

### Ethics statements

In the animal studies animals had free access to food and water and they were housed and treated following the conditions approved by Uppsala Ethical Committee on Animal Experiments, which also approved the experimental protocol (approval C32/3 from 19.03.2003 and C246/10 from 29.09.2010). All patient samples were obtained following approval of the Ethics Committee of Uppsala University (Application Dnr Ups 02-330) and the Ethics Committee of Karolinska Institutet (Application Dnr Ki 02-254). Patients gave written informed consent for the sample collection. The study involving human biopsy samples was conducted in accordance with the Declaration of Helsinki.

### Tissue samples

Constructs and mice used in this study have been previously described [18]. From archival material, a diverse panel of mouse brain specimens was selected. The panel includes *Ntv-a* and *Gtv-a* strains of *Ink4a−/−* and *Arf−/−* background. Tumors were induced by RCAS virus encoding a combination of *KRas+Akt* [17].

Human tissue samples were obtained from Uppsala Biobank material. Both high- and low-grade tumors had been graded based on the WHO classification by experienced neuropathologists.

### Mouse glioma cell culture

Mouse glioma cell culture 3074A was established in *Gtv-a Arf−/−* mouse [18]. Glioma was induced by RCAS virus encoding a combination of *KRas+Akt* [17].

The injected mouse was euthanized when showing any sign of sickness, but at the latest at 12 weeks of age. The brain was collected under aseptic conditions and a coronal section was made at the injection site and one part was collected and embedded in paraffin post formalin fixation, whereas the other part was minced and dissociated for culturing. The mouse cell line was cultured in Dulbeco's Modified Eagle's Medium (DMEM) (Sigma Aldrich, MO, USA), supplemented with 10% fetal bovine serum (FBS) (Invitrogen, Carlsbad, CA), 4 mM L-glutamine and 100 units/ml penicillin and 0.1 mg/ml streptomycin (Sigma Aldrich). The expression of *KRas+Akt* in primary glioma cells was confirmed and corresponding haematoxylin-stained tissue was ranked as grade III with susceptibility of developing into a grade IV tumor.

### BMMC culture

Bone-marrow cells from *Gtv-a* wt mice were obtained from femura and tibia by flushing the bones with 2.5 ml of PBS. The cells were cultured in DMEM, supplemented with 10% heat-inactivated FBS, 60 µg/ml penicillin, 50 µg/ml streptomycinsulfate, 2 mM L-glutamine and 30% WEHI-3B-conditioned media (which contains IL-3). The cells were kept at a concentration of 0.5–1×10^6^ cells/ml with weekly changes of medium. All cells were grown at 37°C with 5% CO2.

### In vitro chemotaxis assay

All chemotaxis experiments were carried out in a 24-well culture plates using hanging inserts with a 5 µm PET membrane (Millipore (Billerica, MA)) where 10^5^ mast cells were placed. Conditioned medium was obtained from confluent 3074a mouse glioma cell culture seeded 72 hours prior to the experiment. It was subsequently added to the lower wells and DMEM supplemented with 10% FBS, 4 mM L-glutamine, 100 units/ml penicillin and 0.1 mg/ml streptomycin was used as a negative control. For neutralization experiments, CXCL12 neutralizing antibody (250 ng/ml, R&D Systems (Abingdon, UK)) was incubated with the conditioned medium for 30 minutes at room temperature. In order to block the receptor on MC surface, CXCR4 antibody (100 µg/ml, R&D Systems) was incubated with MCs for 60 minutes at room temperature. Control samples were incubated under same conditions with a matching isotype nonspecific antibody (mouse monoclonal IgG_1_ and rat monoclonal IgG_2B_, respectively, R&D Systems) diluted to the same concentration. The experiments were performed in triplicate.

### Immunohistochemistry and -fluorescence

Formalin-fixed, paraffin-embedded 6 µm thick tissue sections were fixated onto glass slides. Thereafter, the sections were deparafinized (in xylene over night, in fresh xylene for 1 h on a rocking table followed by 2×5 min incubations in 100% EtOH, 95% EtOH, 80% EtOH, distilled H_2_O) and subjected to pressure boiling for antigen retrieval in antigen unmasking solution (Vector Labs, Burlingame, CA).

Immunohistochemistry was performed using the UltraVision LP detection System (Thermo Fisher Scientific, CA) in accordance with the manufacturer's instructions. Briefly, after antigen retrieval the slides were washed in PBS-T (containing 0.05% Tween (Sigma Aldrich, MO, USA)) and incubated with hydrogen peroxidase block. Ultra V block was subsequently applied. Primary antibody used included anti-mouse SCF (1∶100, Millipore) and anti-human tryptase (1∶200, Santa Cruz (Santa Cruz, CA)), mMCP-6, CPA, mMCP-1, mMCP-2 (1∶200, Antisera raised in rabbit were as described [42]. They were diluted in 5% normal goat serum containing PBS-T and incubated over night at 4°C. Primary antibody diluted in 5% normal goat serum containing PBS-T was applied over night at 4°C, followed by primary antibody enhancer. Slides were incubated with HRP polymer and the signal was visualized using freshly prepared DAB plus chromogen and substrate mix. Between all the steps described above, the slides were thoroughly washed in PBS-T. After the final step, the slides were washed in distilled H_2_O, counterstained with hematoxylin and mounted using Immu-mount (Thermo Fisher Scientific, CA). Pictures were taken using a Leica brightfield microscope.

For immunofluorescence staining, slides were rinsed in PBS, blocked in 5% milk-containing PBS-Tx (supplemented with 0.2% Triton-X 100 (Sigma Aldrich, MO)) for 1 hour, followed by over night incubation (4°C) with the primary antibody diluted in the blocking solution in accordance with producer's guidelines. The following antibodies with the specific dilutions were used: mMCP-6 (1∶200, antiserum raised in rabbit as described previously), anti-mouse Ki-67 (1∶200, DakoCytomation (Glostrup, Denmark), anti-mouse CXCR4 (1∶50, R&D Systems), anti-human CXCR4 (1∶200, Abcam (Cambridge, UK)), anti-mouse CXCL12 (1∶60, R&D Systems), anti-human CXCL12 (1∶100, R&D Systems), anti-human tryptase (1∶250, Santa Cruz (Santa Cruz, CA), anti-mouse CD31 (1∶50, Santa Cruz). The slides were subsequently incubated with appropriate secondary antibody for up to 1 hour and mounted in DAPI (1∶5000) containing Immu-mount. In between the incubations, slides were washed in PBS-Tx. All secondary antibodies were purchased from Invitrogen (Carlsbad, CA) or Jackson ImmunoResearch (West Grove, PA). Pictures were taken using Zeiss 510 META confocal microscope and Zen software (version 5.0, 2008). Where applicable, maximum intensity projection was performed on z-stack images.

Chloroacetate esterase staining was performed as described previously [43].

The quantification of Ki-67 positive MCs was determined from four representative tumors each from *Ntv-a Arf−/−* and *Gtv-a Arf−/−* mice. The percentage of Ki-67-positive nuclei out of 100 randomly selected MCs was calculated.

The quantification of all images was performed by ImageJ software.

The number of MCs per square millimeter was counted in MHb in non-tumor, MHb of tumor-contained sample and in tumor itself of mouse tissues from different genetic backgrounds. The number of MCs in human tumor samples was counted per square millimeter.

### Statistical analysis

Where applicable, quantified data are presented as mean ± SD. To estimate statistical significance, Student's unpaired two-tailed t-test was used.
